# Successful Management of a Completely Disconnected Bile Leak After Hepatectomy Using Endoscopic Ultrasound‐Guided Transluminal Drainage of a Biloma: A Case Report

**DOI:** 10.1002/deo2.70459

**Published:** 2026-10-22

**Authors:** Tatsuki Tanaka, Kohsaku Ohnishi, Hiroshi Yukimoto, Akino Okamoto, Takafumi Tanimoto, Soichiro Mori, Motohiro Hirao, Masanori Tsujie, Atsushi Hosui, Naoki Hiramatsu

**Affiliations:** ^1^ Department of Gastroenterology and Hepatology Osaka Rosai Hospital Sakai Japan; ^2^ Department of Diagnostic Radiology Osaka Rosai Hospital Sakai Japan; ^3^ Department of Gastroenterology Surgery Osaka Rosai Hospital Sakai Japan

**Keywords:** bile leak, endoscopic drainage, endoscopic ultrasonography, hepatectomy, transmural drainage

## Abstract

Post‐hepatectomy bile leak is a common postoperative complication. It usually resolves spontaneously but may be refractory. In cases of completely disconnected bile ducts, endoscopic retrograde cholangiopancreatography (ERCP)–guided drainage is often technically difficult, and invasive additional procedures may be required. We report a completely disconnected bile leak after hepatectomy successfully treated with endoscopic ultrasound‐guided transluminal drainage (EUS‐TD) after unsuccessful EUS‐guided biliary drainage (EUS‐BD). A 78‐year‐old woman underwent anterior sectionectomy for recurrent hepatocellular carcinoma and developed persistent bile leakage. ERCP showed complete disruption of the right posterior sectoral bile duct, and guidewire passage was impossible. EUS‐BD failed because of the nondilated bile duct; therefore, the bile leak‐associated infected biloma was drained through an endoscopically created route under EUS guidance. The infected biloma showed significant shrinkage, and the patient was discharged without complications. EUS‐TD may represent an alternative therapeutic option for completely disconnected bile leaks when EUS‐BD is technically difficult.

## 1 Introduction

Bile leak is a common complication after hepatectomy that occurs at a certain frequency and may lead to severe clinical outcomes such as severe infection, biliary peritonitis, and deterioration of liver function. Although completely disconnected bile leaks are often refractory and their management remains challenging, no standard treatment strategy has been established.

When endoscopic retrograde cholangiopancreatography (ERCP) is not feasible, reported options include reoperation, percutaneous transhepatic biliary drainage, bile duct ablation, portal vein embolization, and magnetic compression anastomosis (MCA). However, these treatment options have several limitations, including high invasiveness, deterioration of quality of life, and limited applicability to selected patients. Recently, endoscopic ultrasound‐guided biliary drainage (EUS‐BD) has attracted attention as a minimally invasive alternative for ERCP failure, and its efficacy and safety have been demonstrated in several studies. However, EUS‐BD can be difficult in cases without bile duct dilation, and alternative endoscopic approaches such as EUS‐guided transluminal drainage (EUS‐TD) have been recommended, although the number of cases remains limited.

Here, we report a case of a completely disconnected bile leak after anterior sectionectomy successfully managed with EUS‐TD when EUS‐BD was technically infeasible.

## 2 Case Report

A 78‐year‐old woman with a history of chronic hepatitis C achieved sustained virological response after interferon therapy. During follow‐up, hepatocellular carcinoma was detected in the anterior segment of the liver, and radiofrequency ablation (RFA) was performed twice. Three months later, contrast‐enhanced computed tomography (CE‐CT) revealed recurrence near the site where RFA was performed, and surgical treatment was planned. Preoperative imaging showed no apparent anatomical variation of the right posterior sectoral bile duct.

Anterior sectionectomy with cholecystectomy was performed. A percutaneous drainage tube was placed on the hepatic resection surface. From POD 3, the bilirubin concentration in the drainage fluid increased to 20.3 mg/dL, leading to a diagnosis of postoperative bile leakage. Furthermore, *Enterococcus faecium* was isolated from the drainage fluid, and antibiotic therapy was required. The patient was referred to our department on POD 8 because the bile leak persisted. CE‐CT demonstrated dilation of the right posterior sectoral bile duct with an adjacent fluid collection near the hepatic resection surface (Figure 1).

**FIGURE 1:**
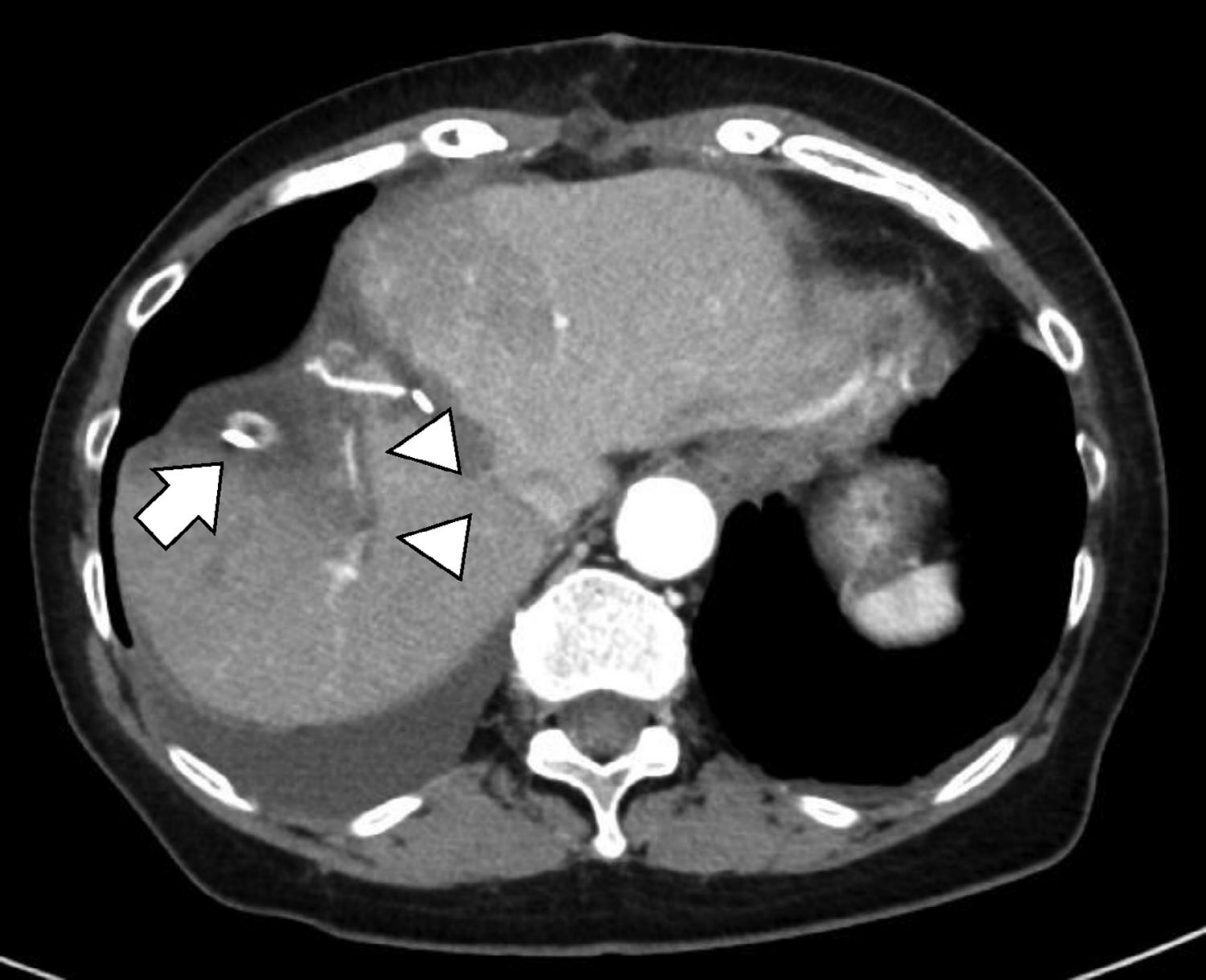
Contrast‐enhanced abdominal computed tomography (CT) performed on postoperative day 8: Status post anterior sectionectomy with cholecystectomy. A fluid collection is observed adjacent to the hepatic resection margin, suspicious for a known postoperative bile leak. A percutaneous drainage catheter is in place within the collection. Compared with the previous examination, the right posterior sectoral bile duct is slightly dilated and appears in close proximity to the fluid collection near the hepatic hilum. **Arrow**, percutaneous drainage catheter; **arrowhead**, intrahepatic bile duct of the posterior segment.

ERCP on POD 9 demonstrated complete interruption of the right hepatic duct. Guidewire passage across the disrupted site was impossible, and a completely disconnected bile leak was diagnosed (Figure 2a). An endoscopic nasobiliary drainage tube was placed in the common bile duct, and careful follow‐up was performed; however, no reduction in bile output from the percutaneous drain was observed. Magnetic resonance cholangiopancreatography and drip‐infusion cholangiography‐CT (DIC‐CT) revealed contrast leakage from the hepatic cut surface communicating with the percutaneous drain. In particular, DIC‐CT demonstrated bile leakage connecting the right posterior sectoral bile duct to the biloma containing the percutaneous drainage tube, confirming postoperative bile leakage from the right posterior sectoral bile duct (Figure 2b,c). After multidisciplinary discussion, reoperation was considered. However, a less invasive approach was preferred, and EUS‐BD was scheduled on POD 24.

**FIGURE 2:**
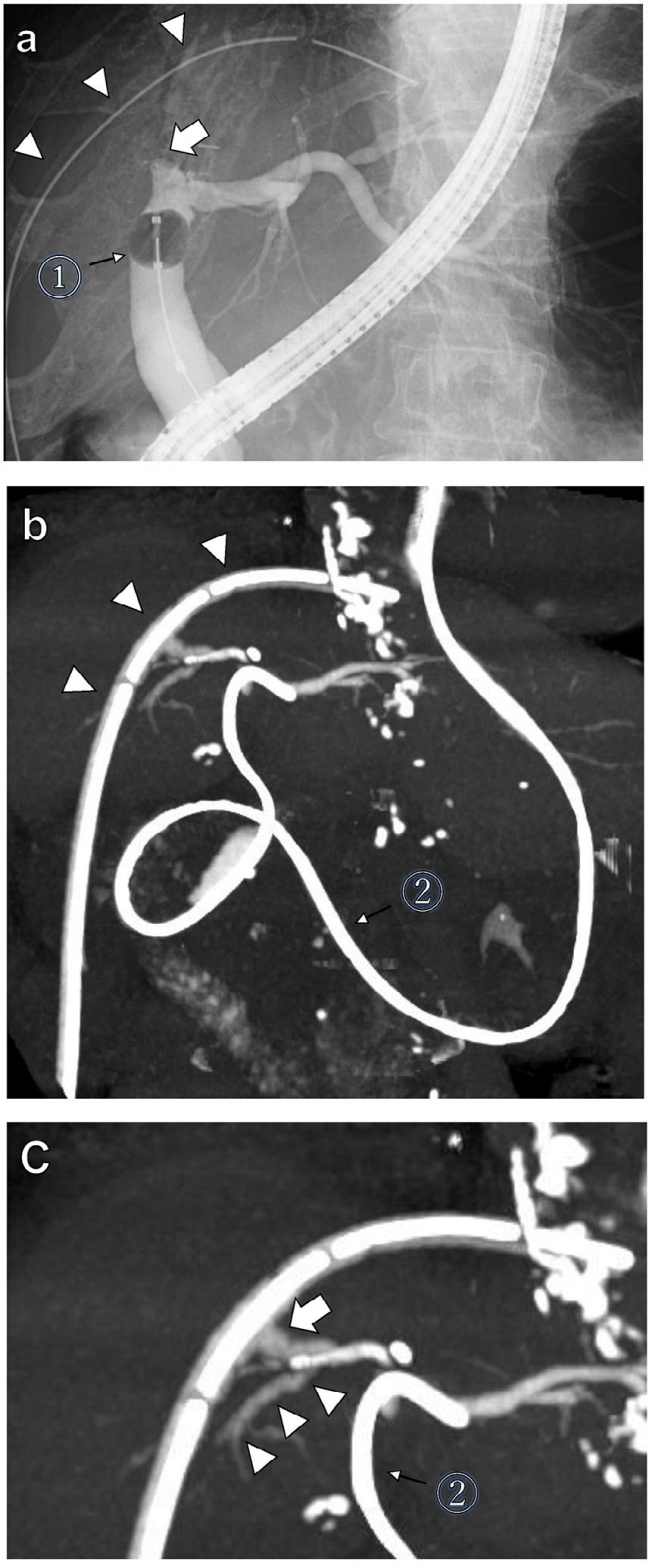
Imaging studies demonstrating a completely disconnected bile leak from the right posterior sectoral bile duct. (a) Endoscopic retrograde cholangiopancreatography (ERCP) performed on postoperative day 9. Cholangiography was performed with occlusion of the distal common bile duct using a balloon catheter (Fusion Quattro; Cook Medical); however, the right hepatic duct was not visualized. **Arrow**, site of interruption of the right hepatic duct; **arrowhead**, percutaneous drainage tube; **①**, balloon catheter. (b) Drip‐infusion cholangiography‐computed tomography (DIC‐CT) performed on postoperative day 20. **Arrowhead**, percutaneous drainage tube; **②**, endoscopic nasobiliary drainage tube (ENBD tube). (c) Magnified DIC‐CT performed on postoperative day 20. Bile leakage was observed from the right posterior sectoral bile duct into the biloma containing the percutaneous drainage tube, confirming postoperative bile leakage from the right posterior sectoral bile duct. **Arrow**, bile leakage; **arrowhead**, the right posterior sectoral bile duct; **②**, ENBD tube.

EUS was performed using a linear echoendoscope (GF‐UCT260; Olympus). The right posterior sectoral bile duct was visualized from the duodenal bulb by injecting contrast through the percutaneous drain (Figure 3a). However, the bile duct diameter was only 1.7 mm. Puncture with a 22‐gauge needle (SonoTip TopGain; Medi‐Globe) was unsuccessful, and EUS‐BD was abandoned.

**FIGURE 3:**
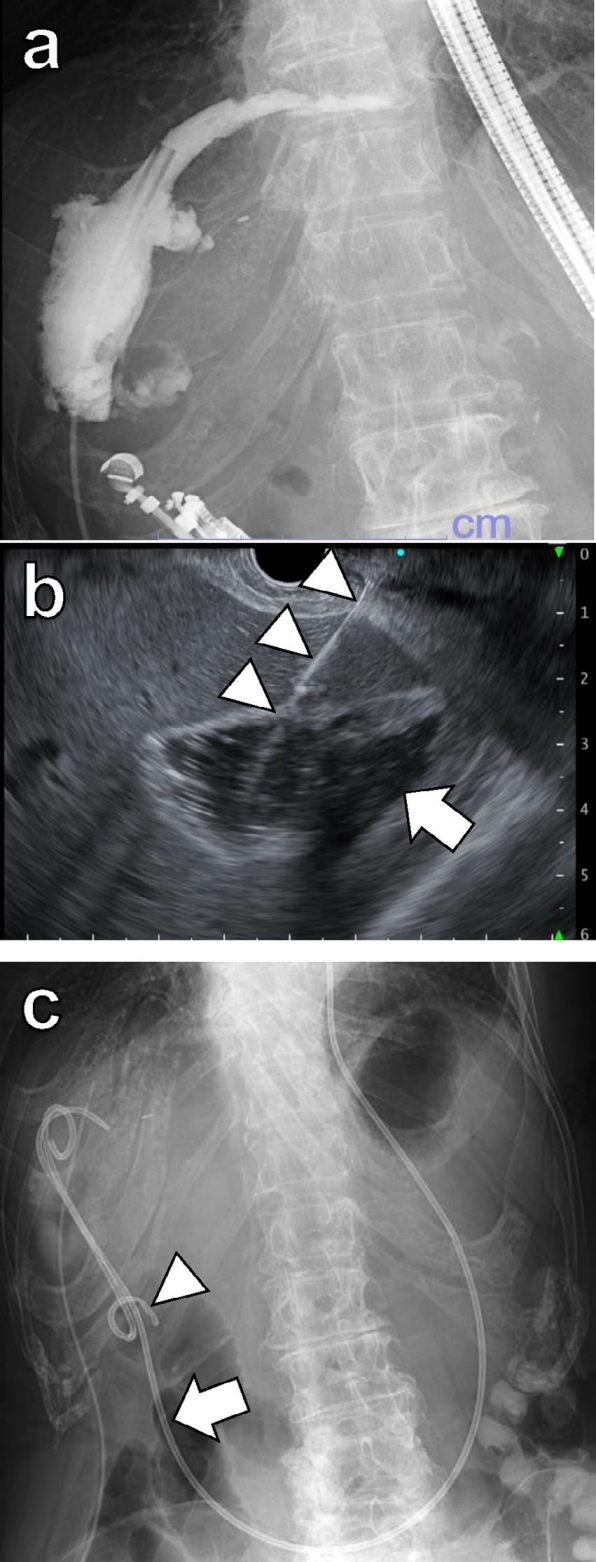
Endoscopic ultrasound‐guided transluminal drainage (EUS‐TD) performed on postoperative day 24. (a) Contrast medium was injected through the percutaneous drainage tube, and EUS visualized the bile leak–associated infected biloma. (b) EUS identified the infected biloma, and a 22‐gauge disposable aspiration needle punctured the biloma. **Arrow**, the infected biloma; **arrowhead**, aspiration needle. (c) Abdominal radiograph obtained after placement of tubes. **Arrow**, transnasal drainage catheter; **arrowhead**, double‐pigtail plastic stent.

Because the bile leak had resulted in an infected biloma, the strategy was changed to EUS‐TD. After injection of 40 mL of saline through the percutaneous drain, the biloma was visualized and punctured with a 22‐gauge needle (EZ Shot 3 Plus; Olympus) (Figure 3b). A 0.018‐inch guidewire (Fielder 18; Olympus) was inserted, and two guidewires were placed using a double‐lumen dilator (MEISSA; Japan Lifeline). The endoscopic created route (ESCR) was dilated with a 4 × 60‐mm balloon catheter (REN TYPE IT; Kaneka Medix), followed by placement of a 7‐Fr, 7‐cm double‐pigtail plastic stent (Advanix J; Boston Scientific) and a 7.5‐Fr transnasal drainage catheter (Flexima; Boston Scientific) (Figure 3c).

Follow‐up computed tomography on POD 25 confirmed appropriate positioning of both drainage tubes. Contrast injection via the percutaneous drain on POD 29 demonstrated flow into the duodenum through the ESCR (Figure 4). The percutaneous drain was gradually downsized and removed on POD 66. The patient was discharged on POD 70.

**FIGURE 4:**
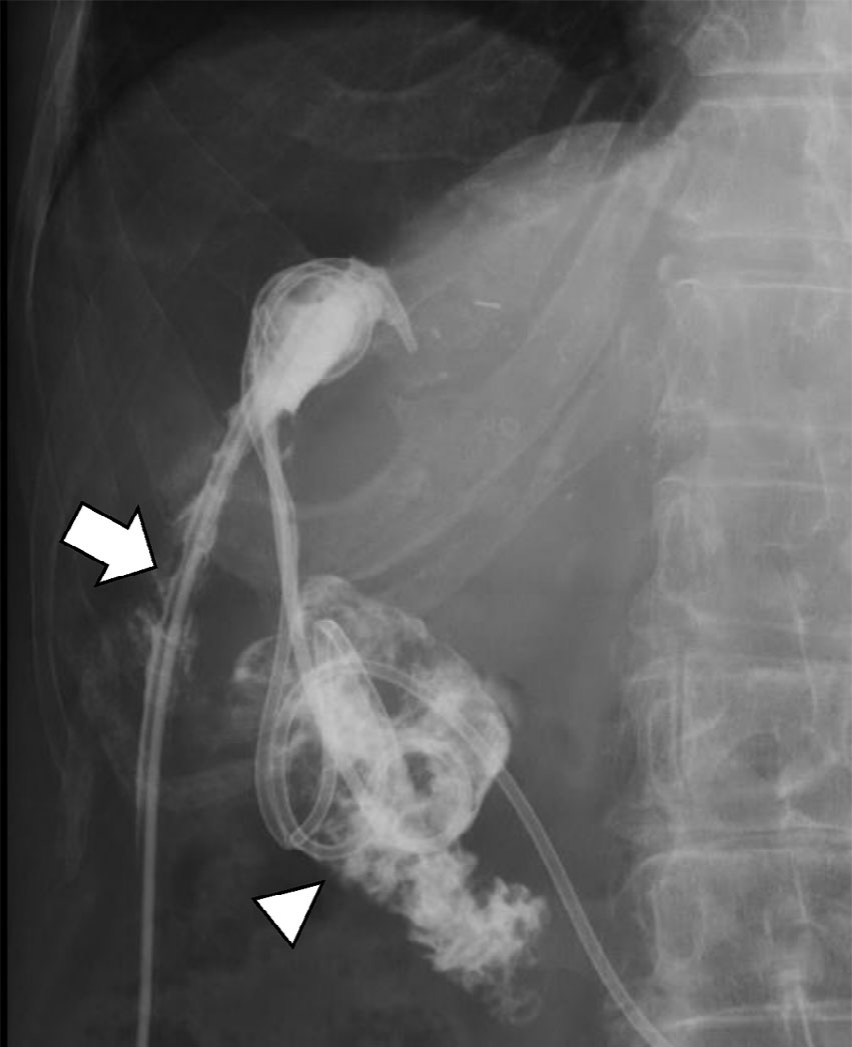
Contrast study performed via the percutaneous drainage tube on postoperative day 29. Contrast medium flowed into the duodenum through the endoscopic created route (ESCR), confirming effective internal drainage. **Arrow**, percutaneous drainage tube; **arrowhead**, contrast flow into the duodenum through the ESCR.

Internal drainage was achieved through the plastic stent placed via the ESCR, with regular stent exchanges thereafter. The patient has been followed for 8 months after EUS‐TD. The plastic stent was exchanged twice at intervals of 3–4 months and remains in place. No recurrence of biloma, cholangitis, or other biliary complications has been observed during the follow‐up period.

## 3 Discussion

According to the International Study Group of Liver Surgery (ISGLS), bile leakage after hepatectomy is defined as drainage fluid with a bilirubin concentration at least three times higher than the serum bilirubin level persisting beyond POD 3, or cases requiring surgical or radiological intervention [1]. Severity is classified into Grade A (no treatment required), Grade B (treatment without re‐laparotomy), and Grade C (re‐laparotomy required) [1]. Nagano et al. further classified bile leaks into central (communicating) and peripheral (disconnected) types [2], which is important for determining management strategies.

While most communicating bile leaks resolve with peritoneal drainage or transpapillary biliary stenting [3], disconnected bile leaks are refractory to standard endoscopic treatment because the bile duct continuity is disrupted, requiring alternative approaches [4]. Preoperative imaging showed no apparent anatomical variation of the right posterior sectoral bile duct, suggesting that anatomical variation was unlikely to have contributed to the bile leak in this case.

Reported non‐endoscopic treatments include reoperation, bile duct ablation, portal vein embolization, transarterial embolization, and Magnetic compression anastomosis (MCA) [3, 5, 6, 7]. Reoperation is invasive and associated with high mortality [5]. Ethanol‐based bile duct ablation may cause irreversible tissue damage and serious systemic complications from inadvertent intravascular injection [6] and is not covered by insurance in Japan.

MCA is another potential treatment for complete biliary disconnection. MCA enables restoration of biliary continuity by creating an anastomosis between the disconnected bile ducts using magnets placed via combined percutaneous and endoscopic approaches [7]. Its major advantage is the restoration of anatomical biliary continuity without reoperation. However, MCA requires access to both sides of the disconnected bile duct and anatomical conditions that allow adequate approximation of the magnets. In the present case, the disconnected right posterior sectoral bile duct was small and not dilated, making direct access to the isolated duct technically difficult. These anatomical features would also have made MCA technically challenging. Percutaneous transhepatic biliary drainage was also considered; however, direct puncture of the nondilated right posterior sectoral bile duct, which measured only 1.7 mm in diameter, was considered technically difficult.

Recently, EUS‐guided drainage techniques have been reported as alternative endoscopic treatments [8]. EUS‐BD enables direct biliary access and internal drainage after failed ERCP but may be challenging in patients with altered anatomy or nondilated bile ducts. In such situations, EUS‐TD may represent another therapeutic option.

In this case, after unsuccessful ERCP and EUS‐BD, we selected EUS‐TD to drain the bile leak‐associated infected biloma. This approach achieved effective infection control and allowed removal of the percutaneous drain. During the 8‐month follow‐up period, the plastic stent was exchanged twice and remains in place, with no recurrence of biloma, cholangitis, or other biliary complications. Although periodic stent exchange is still required, these findings suggest that EUS‐TD can provide sustained internal drainage in this patient. Further follow‐up is needed to determine whether the stent can eventually be removed without recurrence.

Although EUS‐TD has been reported for postoperative intra‐abdominal abscesses, reports describing EUS‐TD for postoperative bile leakage remain limited [9, 10]. This case suggests that EUS‐TD may be a useful minimally invasive option for completely disconnected bile leaks when direct biliary access is not feasible.

In conclusion, we report a case of a completely disconnected bile leak after hepatectomy successfully managed with EUS‐TD. EUS‐TD may represent a useful minimally invasive treatment option for completely disconnected bile leaks when direct biliary access is not feasible. Further studies are warranted to clarify its efficacy and safety.

## Author Contributions

T.T.: endoscopic procedures, clinical management, literature research, Writing – original draft, and writing – review and editing; K.O.: treatment planning and coordination, endoscopic procedures, clinical management, and writing – review and editing; H.Y.: imaging data acquisition and analysis, and writing – review and editing; A.O.: endoscopic procedures, clinical management, and writing – review and editing; T.T.: endoscopic procedures, clinical management, and writing – review and editing; S.M.: treatment planning and coordination, surgical procedures, clinical management, and writing – review and editing; M.H.: endoscopic procedures support, clinical management, and writing – review and editing; M.T.: management planning and multidisciplinary coordination, final treatment decision, surgical procedures, clinical management, and writing – review and editing; A.H.: overall clinical supervision, final treatment decision, writing – review and editing, and co‐guarantor of the entire case report; N.H.: overall clinical supervision, writing – review and editing, and guarantor of the entire case report.

## Conflicts of Interest

The authors declare no conflicts of interest.

## Funding

The authors have nothing to report.

## Ethics Statement

Informed consent for publication, including radiologic and endoscopic images, was obtained from the patient.
